# O9.1. CANNABIS AND OTHER SUBSTANCE USE DISORDERS PREDICT CONVERSION FROM SCHIZOTYPAL DISORDER TO SCHIZOPHRENIA

**DOI:** 10.1093/schbul/sby015.245

**Published:** 2018-04-01

**Authors:** Carsten Hjorthoj, Nikolai Albert, Merete Nordentoft

**Affiliations:** 1 Copenhagen University Hospital, Mental Health Center Copenhagen; 2 Mental Health Center Copenhagen; 3 Mental Health Centre Copenhagen, The Lundbeck Foundation Initiative for Integrative Psychiatric Research (iPSYCH)

## Abstract

**Background:**

Schizotypal disorder has been linked to conversion to schizophrenia, with a quarter to half of patients converting over a course of two to five years. Substance use disorders are common in patients with schizotypal disorder, and cannabis has been shown to be a risk factor for developing schizotypal disorder. Cannabis has also been linked to an increased risk of schizophrenia. Other substance use disorders, in particular alcohol, may also increase risk of later schizophrenia. These previous results suggest that substance use, in particular cannabis, may predict conversion to schizophrenia in people with schizotypal disorder.

**Methods:**

We used the nationwide, unselected Danish registers. The study population was all people born since 1981 in Denmark with incident diagnosis of schizotypal disorder, without previous diagnosis of schizophrenia. Information on substance use disorders was combined from five different registers. Cox regression using time-varying information on substance use disorders and antipsychotic medication, and adjusted for parental history of mental disorders, sex, birth year, and calendar year were used to estimate hazard ratios (HR) and 95% confidence intervals (CI).

**Results:**

After two years, 16.3% (95% CI 14.8%-17.8%) of the people with schizotypal disorder had converted to schizophrenia. After 20 years, the conversion rate was 33.1% (95% CI 29.3–37.3) overall, and 58.2% (95% CI 44.8%-72.2%) in those with cannabis use disorders. In fully adjusted models, any substance use disorder predicted conversion to schizophrenia (HR=1.34, 95% CI 1.11–1.63). Dividing by substances, cannabis use disorders (HR=1.30, 95% CI 1.01–1.68), amphetamine use disorders (HR=1.90, 95% CI 1.14–3.17), and opioid use disorders (HR=2.74, 95% CI 1.38–5.45) predicted conversion to schizophrenia. These associations were not explained by concurrent use of antipsychotic medication, functional level before incident schizotypal disorder, or parental history of mental disorders.

**Discussion:**

Substance use disorders, in particular cannabis, amphetamines, and opioids, are important predictors of conversion from schizotypal disorder to schizophrenia. However, conversion rates are high even in those without substance use disorders, indicating a need for both universal and substance-targeted prevention in people with schizotypal disorder.

